# Enhancing Antibacterial Dental Matrices: Balancing Antibacterial Activity and Mechanical Properties Through Quaternary Ammonium UDMA Analogues

**DOI:** 10.3390/polym18030426

**Published:** 2026-02-06

**Authors:** Marta Chrószcz-Porębska, Alicja Kazek-Kęsik, Izabella Ślęzak-Prochazka, Grzegorz Chladek, Izabela Maria Barszczewska-Rybarek

**Affiliations:** 1 Chair of Drug and Cosmetics Biotechnology, Faculty of Chemistry, Warsaw University of Technology, Noakowskiego 3 Street, 00-664 Warsaw, Poland; marta.porebska@pw.edu.pl; 2 Department of Inorganic Chemistry, Analytical Chemistry and Electrochemistry, Faculty of Chemistry, Silesian University of Technology, Krzywoustego 6 Street, 44-100 Gliwice, Poland; alicja.kazek-kesik@polsl.pl; 3 Biotechnology Centre, Silesian University of Technology, Krzywoustego 8 Street, 44-100 Gliwice, Poland; izabella.slezak-prochazka@polsl.pl; 4 Materials Research Laboratory, Faculty of Mechanical Engineering, Silesian University of Technology, 18a Konarskiego Str., 41-100 Gliwice, Poland; grzegorz.chladek@polsl.pl; 5 Department of Physical Chemistry and Technology of Polymers, Faculty of Chemistry, Silesian University of Technology, Strzody 9 Street, 44-100 Gliwice, Poland

**Keywords:** dental materials, quaternary ammonium compounds, antibacterial dental matrices, physico-mechanical properties, photopolymerization

## Abstract

The research hypothesis was that adjusting the content of the quaternary ammonium urethane dimethacrylate monomer bearing an N-dodecyl substituent (QAUDMA-12) would yield dental matrices with high antimicrobial activity, good biocompatibility, and favorable physicochemical properties. The research hypothesis was verified for six Bis-GMA, TEGDMA, and UDMA copolymers containing from 2.5 to 40 wt.% QAUDMA-12 by determining their degree of conversion, hardness, flexural properties, water behavior, antimicrobial activity against *Staphylococcus aureus*, *Staphylococcus epidermidis*, *Escherichia coli*, *Candida albicans*, and cytotoxicity towards L929 mouse fibroblast cells. The research hypothesis was confirmed. Copolymers containing less than 30 wt.% QAUDMA-12 exhibited favorable polymerization efficiency, water sorption and solubility, and mechanical properties comparable to those of conventional Bis-GMA/TEGDMA systems. At the same time, they showed no cytotoxic effects toward mouse fibroblast cells. The results of antimicrobial tests show that the minimum QAUDMA-12 concentration providing sufficient antimicrobial activity was 20 wt.%. Therefore, it can be concluded that the 20 wt.% concentration of QAUDMA-12 makes it possible to obtain dental matrices that are non-toxic, exhibit antimicrobial activity, and possess the desired physico-mechanical performance.

## 1. Introduction

In recent decades, dental composite restorative materials (DCRMs) have undergone substantial development [1,2,3]. However, the prevalence of secondary caries remains a persistent challenge in clinical dentistry. This is primarily associated with microbial colonization at the tooth–restoration margin, leading to degradation of the restorative material and demineralization of the surrounding tissues [4,5]. While offering aesthetic and mechanical benefits, DCRMs inherently lack antibacterial properties, necessitating the exploration of novel materials that can serve both structural and antimicrobial roles [3].

In this context, incorporating antimicrobial quaternary ammonium compounds (QACs) into dental composite matrices has garnered much attention [6]. QACs, particularly in the form of quaternary ammonium methacrylate (QAM) monomers—bearing polymerizable methacrylate groups and quaternary ammonium moieties—have been extensively studied for dental applications [7,8,9]. Their incorporation into dental matrices has demonstrated enhanced antibacterial efficacy against oral pathogens, notably *Streptococcus mutans*, a key etiological agent in secondary caries formation. However, limitations associated with QAM-based dental matrices, including elevated cytotoxicity and compromised mechanical properties, remain a concern.

**Figure 1:**
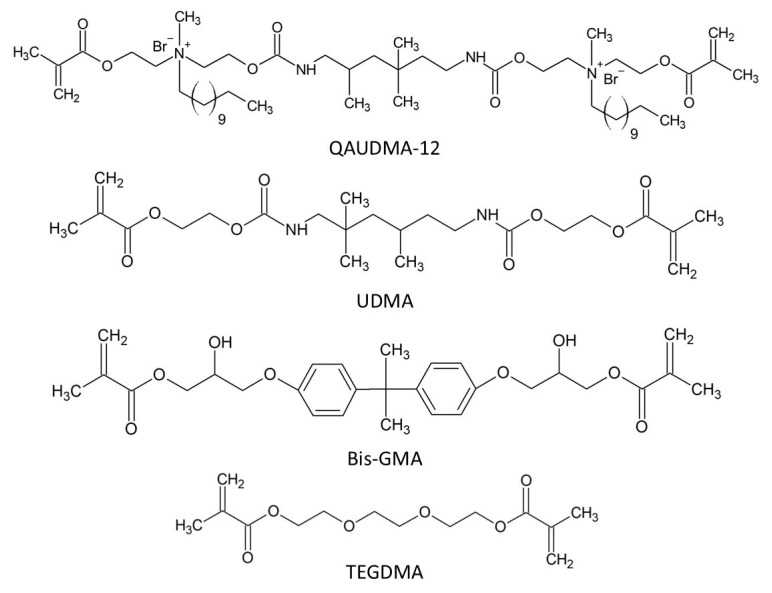
Dimethacrylates used in the copolymers tested. Reprinted with permission from Ref. [13]. Multidisciplinary Digital Publishing Institute, 2023.

To meet these challenges, we have developed six quaternary urethane ammonium dimethacrylates (QAUDMA-m, where the “m” denotes the length of the N-alkyl substituent, precisely the number of carbon atoms). Their structural precursor was the urethane dimethacrylate monomer (UDMA) (Figure 1) [10]. These new monomers were incorporated into resin matrices based on standard dental dimethacrylates—Bis-GMA and TEGDMA (Figure 1) [11,12,13,14,15].

The resulting copolymers exhibited substantial antimicrobial activity, effectively inhibiting the adhesion of *Staphylococcus aureus* [11,14], *Escherichia coli* [11,14], and *Candida albicans* [14]. Moreover, preliminary mechanical characterization revealed that the formulations containing QAUDMA-m retained flexural strength and modulus values comparable to, or slightly lower than, those of the standard dental formulations [15]. The length of the N-alkyl substituent significantly influenced both antibacterial efficacy and mechanical performance, with QAUDMA-12 emerging as the most promising candidate. Compared to the copolymers containing QAUDMA-m monomers with shorter alkyl chains (QAUDMA-8, QAUDMA-10), QAUDMA-12 showed slightly higher degree of conversion (*DC*) [11,15], a higher water contact angle (*WCA*) [11,13], lower water sorption (*WS*) and solubility (*SL*) [12,13], lower hardness (*HB*) [12,15], lower flexural strength (*FS*) and modulus (*E*) [12,15], slightly lower antibacterial activity against *S. aureus* and similar antibacterial activity against *E. coli* [11,15], similar antifungal activity against *C. albicans* [14], and lower cytotoxicity [14]. Compared to the copolymers based on QAUDMA-m monomers with longer N-alkyl chains (QAUDMA-14, QAUDMA-16, QAUDMA-18), QAUDMA-12 showed lower *DC* [11,15], lower *WCA* [11,13], higher *WS* and *SL* [12,13], higher *HB* [12,15], higher *FS* and *E* [12,15], lower antibacterial activity [11,15], similar antifungal activity [14] and similar cytotoxicity [14]. QAUDMA-8 and QAUDMA-10 were excluded from further studies because their copolymers exhibited water behavior outside the standards for dental materials. QAUDMA-14, QAUDMA-16, and QAUDMA-18, although their copolymers exhibited appropriate physicochemical and mechanical properties, were excluded from further research because they did not provide sufficient antimicrobial activity. QAUDMA-12 monomer offers the best overall balance of mechanical and antimicrobial properties. Nonetheless, due to the hydrophilic nature of the quaternary ammonium groups, QAUDMA-12-containing copolymers exhibited increased *WS* and *SL* [12,13], warranting further optimization for long-term durability.

Therefore, in this study, a series of copolymers enriched with QAUDMA-12 was formulated to optimize its content within the dental matrices. The research hypothesis was that adjusting the QAUDMA-12 content would enable the development of antibacterial dental matrices with an optimal balance of microbiological efficacy, biocompatibility, and physicochemical properties suitable for clinical application. The research hypothesis was verified for copolymers of 40 wt.% Bis-GMA, 20 wt.% TEGDMA and varied complementarily from 0 to 40 wt.% concentration of QAUDMA-12 and UDMA by determining their (i) degree of conversion, (ii) mechanical properties (flexural strength, flexural modulus, hardness), (iii) water sorption and solubility, (iv) antimicrobial activity against *S. aureus*, *Staphylococcus epidermidis*, *E. coli*, *C. albicans*, and (v) cytotoxicity to the L929 mouse fibroblast cell line.

## 2. Materials and Methods

### 2.1. Sample Preparation

Six resin compositions were formulated by combining bisphenol A glycerolate dimethacrylate (Bis-GMA, Sigma Aldrich, St. Louis, MO, USA), urethane-dimethacrylate (UDMA, Sigma Aldrich, St. Louis, MO, USA), triethylene glycol dimethacrylate (TEGDMA, Sigma Aldrich, St. Louis, MO, USA), and a quaternary ammonium urethane-dimethacrylate analogue with N-dodecyl substituent (QAUDMA-12), synthesized according to the previously described procedure [10]. The components were homogenized by mechanical stirring at 60 °C for 2 h. The Bis-GMA and TEGDMA content was held constant at 40 and 20 wt.%, respectively, while the combined proportion of UDMA and QAUDMA-12 accounted for the remaining 40 wt.%. Within this fraction, the QAUDMA-12 ratio was systematically varied from 2.5 to 37.5 wt.%. The reference composition of 40 wt.% Bis-GMA, 20 wt.% TEGDMA and 40 wt.% UDMA was also prepared (Table 1).

**Figure 2:**
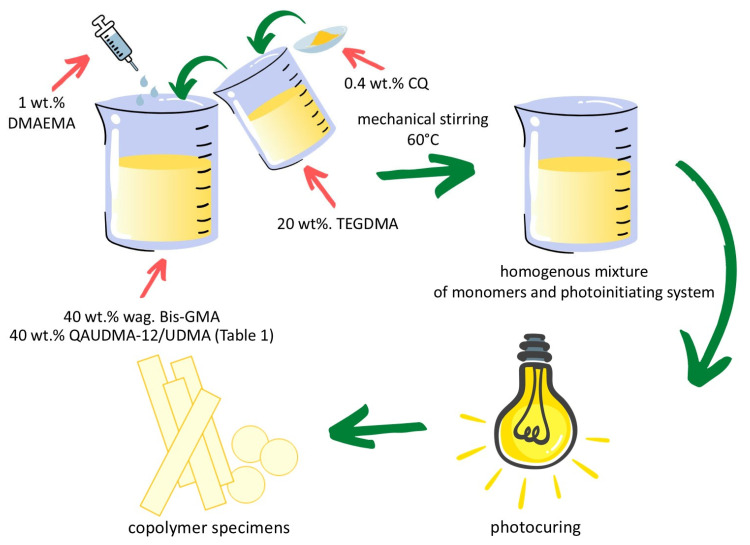
Sample preparation procedure. Reprinted with permission from Ref. [14]. Multidisciplinary Digital Publishing Institute, 2023.

The polymerization process was conducted in square-shaped glass molds (90 mm × 90 mm × 4 mm; length × width × thickness) for mechanical testing and in Teflon disk-like molds (10 mm × 3 mm; diameter × thickness) for antimicrobial and cytotoxicity testing. Monomer compositions enriched with 0.4 wt.% camphorquinone (CQ, Sigma-Aldrich, St. Louis, MO, USA) and 1 wt.% 2-dimethylaminoethyl methacrylate (DMAEMA, Sigma-Aldrich, St. Louis, MO, USA) were placed in molds, covered with PET film, and exposed to UV-VIS light (280–700 nm) using an Ultra Vitalux 300 lamp (Osram, Munich, Germany, radiant exitance of 2400 mW/cm^2^) at room temperature for 1 h (Figure 2).

### 2.2. Degree of Conversion

The degree of conversion (*DC*) was determined from Fourier Transform Infrared Spectroscopy (FT-IR) spectra of liquid monomer compositions and cured polymers.

Samples were analyzed as KBr pellets. A thin layer of the liquid composition was placed between two KBr pellets, whereas cured samples were ground to a grain size of less than 25 μm and homogeneously mixed with KBr. FT-IR spectra were recorded utilizing a Spectrum Two (Perkin-Elmer, Waltham, MA, USA) spectrometer, with 128 scans at a resolution of 1 cm^−1^.

The *DC* was then calculated according to the following equation:

(1) $$DC\;\left(\%\right)=\left(1-\frac{{\left(\frac{A_{C=C}}{A_{Ar}}\right)}_{polymer}}{{\left(\frac{A_{C=C}}{A_{Ar}}\right)}_{liquid\;composition}}\right)\times 100,$$

where:

*A_C=C_*—the absorption intensity of the C=C stretching vibrations in the methacrylate group, at 1637 cm^−1^;
*A_Ar_*—the absorption intensity of the C=C skeletal aromatic stretching vibrations, at 1610 cm^−1^.

### 2.3. Mechanical Properties

The hardness (*HB*) was determined utilizing a VEB Werkstoffprüfmaschinen apparatus (Leipzig, Germany) in accordance with ISO 2039-1 [16]. Disks measuring 40 mm × 4 mm (diameter × thickness) were used in experiments.

The *HB* value was calculated using the following equation:

(2) $$HB\;\left(MPa\right)=\frac{F_{m}\frac{0.21}{\left(h\;-\;h_{r}\right)\;+\;0.21}}{\pi dh_{r}},$$

where:

*F_m_*—the applied load;
*d*—the intender diameter (*d* = 5 mm);
*h*—the immersion depth;
*h_r_*—the reduced immersion depth (*h_r_* = 0.25 mm).

Bars measuring 80 mm × 10 mm × 4 mm (length × width × thickness) were used to determine flexural strength (*FS*) and flexural modulus (*E*). The tests were conducted on a universal testing machine (Zwick Z020, Ulm, Germany) in accordance with ISO 178 [17].

*FS* and *E* were calculated according to the following equations:

(3) $$FS\;\left(MPa\right)=\frac{3Pl}{2bd^{2}},$$

(4) $$E\;\left(MPa\right)=\frac{P_{1}l^{3}}{4bd^{3}\delta },$$

where:

*P*—the maximum applied load;
*P*_1_—the load at the selected point of the elastic region of the stress–strain plot;
*l*—the distance between supports (*l* = 64 mm);
*b*—the specimen width (*b* = 10 mm);
*d*—the specimen thickness (*d* = 4 mm);
δ—the deflection of the sample at *P*_1_.

### 2.4. Water Behavior

Water sorption (*WS*) and solubility (*SL*) were assessed in accordance with ISO 4049 [18].

Initially, disks measuring 15 mm × 5 mm (diameter × thickness) were conditioned in a drying oven (POL-EKO, Wodzisław Śląski, Poland) at 100 °C until a stable mass (*m_0_*) was reached, typically after 72 h. Subsequently, they were immersed in distilled water at room temperature for seven days. After incubation, the samples were removed, gently blotted to remove surface moisture, and weighed to obtain the mass after water absorption (*m*_1_). The samples were dried again at 100 °C until a constant final mass (*m*_2_) was recorded. All weight measurements were performed using an analytical balance (XP Balance, Mettler Toledo, Greifensee, Switzerland) with a precision of 0.01 mg.

*WS* and *SL* were calculated according to the following formulas:

(5) WS μgmm3=m1−m0V,

(6) SL μgmm3=m1−m2V

where:

*m*_0_—the initial mass of the dried sample;
*m*_1_—the mass of the sample after immersion in water;
*m*_2_—the mass of the dried sample after water immersion;
*V*—the initial volume of the dried sample.

### 2.5. Antimicrobial Activity

The adhesion of *Staphylococcus aureus* (ATCC 25923), *Staphylococcus* epidermidis (ATCC 12228), *Escherichia coli* (ATCC 25922), and *Candida albicans* (ATCC 2091) to copolymer surfaces was evaluated using 10 mm diameter, 3 mm thick disks. Prior to testing, all sample surfaces were uniformly abraded with sandpaper to ensure consistent surface roughness.

Prepared specimens were immersed in 1 mL of microbial suspension (approximately 5 × 10^6^ CFU/mL) in an appropriate culture medium and incubated at 37 °C for 18 h (incubator POL-EKO, Wodzisław Śląski, Poland). After incubation, the suspension was carefully removed with a Pasteur pipette, and each sample was gently rinsed with 1 mL of sterile distilled water to remove loosely adhered cells. Subsequently, the rinsed specimens were transferred into sterile centrifuge tubes containing 1 mL of sterile water and vortexed for 1 min at 3000 rpm to dislodge adherent microorganisms from the material surfaces. 100 μL aliquots of the obtained suspension were serially diluted in 0.9% NaCl to achieve final concentrations of 0.1%, 0.01%, 0.001%, 0.0001%, 0.00001%, and 0.000001%. Then, 100 μL of each dilution was placed on an agar plate (Sabouraud agar for fungal culture and Müller-Hinton agar for bacterial culture; Diag-Med, Warszawa, Poland). Plates were incubated at 37 °C for 18 h. After incubation, the colony forming units (CFUs) were manually counted under transmitted light to assess microbial adhesion to the copolymer surfaces.

### 2.6. Cytotoxicity

Cytotoxicity was evaluated in accordance with ISO 10993-5 [19] using the L929 mouse fibroblast cell line (ATCC-CCL-1, NCTC clone 929 Areolar Fibroblast Mouse) provided by LGC Standards (Łomianki, Poland).

Disk-shaped copolymer samples measuring 10 mm × 3 mm (diameter × thickness) were carefully polished, cleaned, and immersed in culture medium (1 mL per 3 cm^2^ of specimen surface). Samples were incubated for 72 h at 37 °C with agitation (80 rpm). The resulting extracts were then mixed with fresh culture medium in a 1:1 ratio and transferred to a 96-well plate. Subsequently, suspensions containing 5000 L929 mouse fibroblast cells per well were added and incubated at 37 °C in a 5% CO_2_ atmosphere for 24 h using an IncuSafe CO_2_ incubator (MCO-170AICUVD-PE, PHCbi, Etten-Leur, The Netherlands). Following incubation, 10 µL of Alamar Blue reagent (G-Biosciences, St. Louis, MO, USA) was added to each well. Samples were further incubated for 4 h at 37 °C. Finally, fluorescence intensity was measured using a Varioskan LUX multimode microplate reader (Thermo Scientific, Waltham, MA, USA).

The results were expressed as relative cell viability compared to the control (Tissue Culture Polystyrene (TCPS)).

### 2.7. Statistical Analysis

The results were expressed as the average of five measurements, with the corresponding standard deviation (*SD*). To assess the statistical significance of the results, a nonparametric Kruskal–Wallis test with Mann–Whitney U post hoc test at a significance level (*p*) of 0.05 was performed using Statistica 13.1 (TIBCO Software Inc., Palo Alto, CA, USA).

## 3. Results

In this study, six copolymers of 40 wt.% Bis-GMA, 20 wt.% TEGDMA and 40 wt.% UDMA/QAUDMA-12 with varying ratios from 0 to 40 wt.% (abbreviated by the wt.%QA12) were prepared by photopolymerization (Table 1) and tested for their physico-mechanical and antimicrobial properties as well as cytotoxicity. The sample without QAUDMA-12 served as a reference.

**Figure 3:**
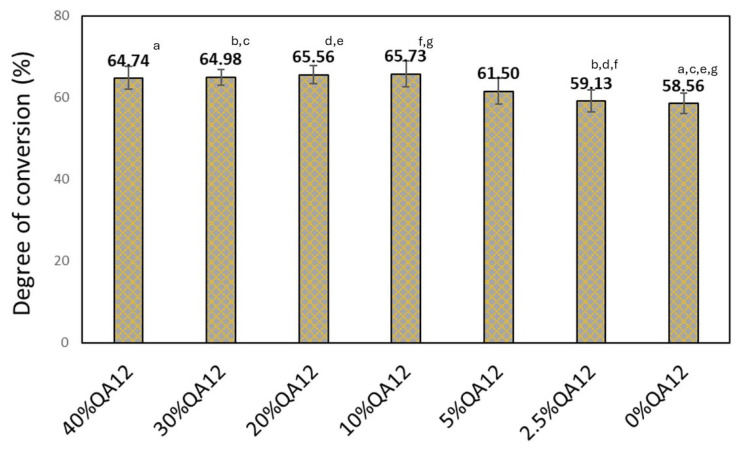
The *DC* of the studied copolymers. Lowercase letters indicate statistically significant (*p* ≤ 0.05) differences (non-parametric Kruskal–Wallis with Mann–Whitney U post hoc test).

**Figure 4:**
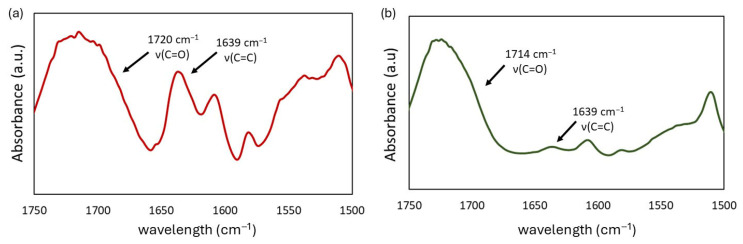
The representative FT-IR spectra of the 20%A12 composition in its uncured (**a**) and cured (**b**) forms.

The degree of conversion (*DC*) in QAUDMA-12-based copolymers ranged from 59.13 to 65.73% (Figure 3 and Figure 4). The *DC* of copolymers with QAUDMA-12 content above 5% was similar (*p* > 0.05), averaging 65.25%. Those values were higher (*p* ≤ 0.05) than the *DC* value of the reference copolymer (*DC* = 58.56%). The remaining QAUDMA-12 containing copolymers showed similar (*p* > 0.05) values to the reference copolymer with an average of 60.32%.

**Figure 5:**
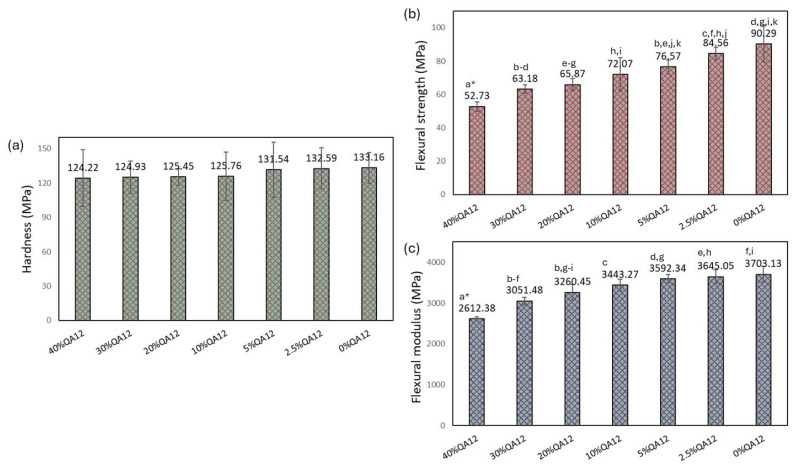
The mechanical properties of the studied copolymers: hardness (*HB*) (**a**), flexural strength (*FS*) (**b**), and flexural modulus (*E*) (**c**). Lowercase letters indicate statistically significant (*p* ≤ 0.05) differences with a column (non-parametric Kruskal-Wallis with Mann-Whitney U post-hoc test). An * indicates that the result was statistically significantly different compared to the results achieved by the remaining samples.

The hardness (*HB*) of QAUDMA-12-based copolymers ranged from 124.22 to 132.59 MPa (Figure 5). Those values were similar to each other (*p* < 0.05) with an average of 12,741 MPa and similar (*p* > 0.05) to the *HB* of the reference copolymer (*HB* = 133.16 MPa). The flexural strength (*FS*) of QAUDMA-12-based copolymers ranged from 52.73 to 84.56 MPa and increased with the decreasing QAUDMA-12 content (Figure 5). The *FS* of copolymers with QAUDMA-12 content higher than 2.5% was significantly lower (*p* ≤ 0.05) than that of the reference copolymer (*FS* = 90.29 MPa). Copolymer containing 2.5 wt.% QAUDMA12 showed a value similar to that of the reference copolymer (*p* > 0.05). The flexural modulus (*E*) of QAUDMA-12-based copolymers ranged from 2612.38 to 3645.05 MPa and increased with the decreasing QAUDMA-12 content (Table 2). The *E* of copolymers with QAUDMA-12 content higher than 10 wt.% was significantly lower (*p* ≤ 0.05) than that of the reference copolymer (*E* = 3703.13 MPa). The remaining QAUDMA-12 containing copolymers showed similar (*p* > 0.05) *E* values to the reference copolymer.

**Figure 6:**
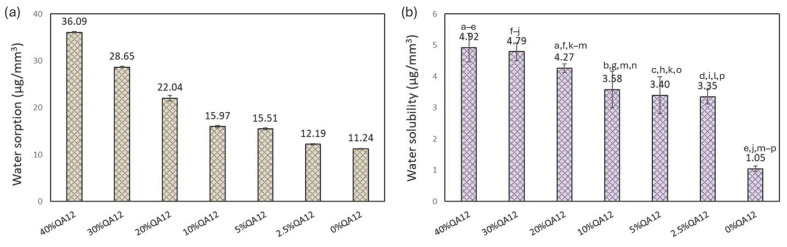
The water sorption (*WS*) (**a**) and solubility (*SL*) (**b**) of the studied copolymers. Lowercase letters indicate statistically significant (*p* ≤ 0.05) differences with a column (non-parametric Kruskal–Wallis with Mann–Whitney U post hoc test). All results for *WS* were statistically significant.

The *WS* of QAUDMA-12-based copolymers ranged from 12.19 to 36.09 µg/mm^3^ and decreased with the decreasing QAUDMA-12 content (Figure 6). All of the obtained values were higher (*p* ≤ 0.05) than that of the reference copolymer (*WS* = 11.24 µg/mm^3^). The *SL* of QAUDMA-12-based copolymers ranged from 3.35 to 4.92 µg/mm^3^ and decreased with the decreasing QAUDMA-12 concentration (Figure 6). All of the obtained values were higher (*p* ≤ 0.05) than that of the reference copolymer (*SL* = 1.05 µg/mm^3^).

Microorganism colony counts on copolymer surfaces increased as QAUDMA-12 content decreased (Table 2). For *S. aureus*, counts ranged from 2.59 to 5.84 log(CFU/mL), which was significantly lower (*p* ≤ 0.05) than the reference value of 6.74 log(CFU/mL). *S. epidermidis* counts on experimental copolymers ranged from 4.48 to 7.22 log(CFU/mL). Copolymers with QAUDMA-12 content above 10 wt.% had significantly fewer adhered bacteria (*p* ≤ 0.05) than the reference copolymer (7.36 log(CFU/mL)). The remaining QAUDMA-12-based copolymers showed similar (*p* > 0.05) values to the reference copolymer. The number of *E. coli* bacteria adhered to QAUDMA-12-based copolymers ranged from 0 to 6.43 log(CFU/mL). The number of bacteria adhered to copolymers with QAUDMA-12 content higher than 5 wt.% was lower (*p* ≤ 0.05) than that of the reference copolymer (6.70 log(CFU/mL)). The remaining QAUDMA-12-based copolymers showed similar (*p* > 0.05) values to the reference copolymer. The number of *C. albicans* fungi adhered to QAUDMA-12-based copolymers ranged from 2.26 to 3.65 log(CFU/mL). Copolymer containing 40 wt.% QAUDMA-12 showed a lower (*p* ≤ 0.05) number of adhered fungi compared to the reference copolymer (4.07 log(CFU/mL)). The remaining QAUDMA-12-based copolymers showed similar (*p* > 0.05) values to the reference copolymer.

**Figure 7:**
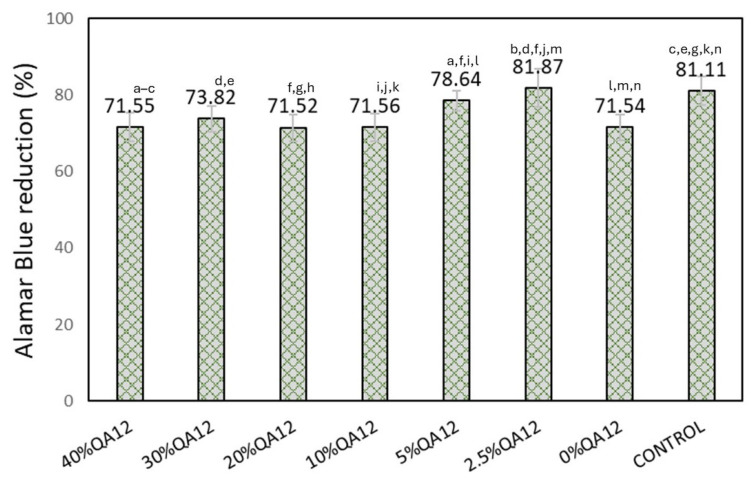
Viability of L929 mouse fibroblast cells after 24 h of incubation. Lowercase letters indicate statistically significant (*p* ≤ 0.05) differences with a column (non-parametric Kruskal–Wallis with Mann–Whitney U post hoc test). The results were expressed as relative cell viability compared to the control (Tissue Culture Polystyrene (TCPS)).

The cell viability of QAUDMA-12-based copolymers ranged from 71.52 to 81.87% (Figure 7). Copolymers with QAUDMA-12 content higher than 5 wt.% showed similar cell viability (*p* > 0.05) to the reference copolymer. The remaining QAUDMA-12-based copolymers showed slightly higher (*p* ≤ 0.05) cell viability than that of the reference copolymer (71.54%).

## 4. Discussion

Incorporating antibacterial properties into dental composites represents a promising strategy to counteract bacterial colonization at the restoration interface. Our earlier studies demonstrated that quaternary ammonium urethane-dimethacrylate monomers (QAUDMA-m) can inhibit bacterial adhesion. However, their incorporation may compromise physico-mechanical properties by reducing hardness and flexural strength and increasing water sorption and solubility.

This study hypothesized that adjusting QAUDMA-12 content would support the development of dental composites with high antimicrobial activity, biocompatibility, and clinically acceptable physicochemical properties. QAUDMA-12 was chosen for optimization because previous studies have shown that its copolymers with commercial dental dimethacrylates offer the best balance of antimicrobial effectiveness and physicochemical properties [14,15]. To test this hypothesis, six copolymers of Bis-GMA, TEGDMA, UDMA, and QAUDMA-12 were synthesized by photopolymerization (Table 1), with QAUDMA-12 content ranging from 40 to 2.5 wt.%. A reference material without QAUDMA-12 was also prepared. The copolymers were evaluated for degree of conversion, mechanical properties, antimicrobial activity, and cytotoxicity.

Table 3 presents a comparative overview of the investigated properties, indicating whether each modified copolymer exhibits higher, lower, or comparable values relative to the reference copolymer.

According to the literature, a degree of conversion (*DC*) above 55% is considered the minimum to ensure the functional integrity and clinical durability of dental restorative materials [20]. A higher *DC* is associated with increased cross-linking density, reduced residual monomer content, and enhanced mechanical strength, all of which contribute to the restoration’s long-term stability and biocompatibility. In the present study, the lowest *DC* value recorded among the QAUDMA-12-based copolymers was 59% (Figure 3), indicating that all tested formulations met the requirements. Consequently, the evaluated materials exhibit sufficient methacrylate conversion to maintain their mechanical and physical properties during clinical use.

Further evaluation of the suitability of QAUDMA-12-based copolymers for use as dental matrix materials was conducted by comparing their mechanical performance with that of a reference copolymer.

Hardness (*HB*) values remained relatively unaffected by the increasing concentration of QAUDMA-12, indicating that introducing urethane-based monomer units, even at elevated levels, does not compromise surface hardness (Figure 5). This stability in *HB* can be attributed to the rigid urethane linkages in QAUDMA-12, which help maintain adequate surface resistance despite possible variations in network architecture [21]. In contrast, a concentration-dependent decrease in flexural strength (*FS*) and flexural modulus (*E*) was observed with increasing QAUDMA-12 content (Figure 5). This decline is likely due to a reduction in crosslink density, resulting from the relatively high molecular weight of QAUDMA-12 compared to UDMA. A less densely crosslinked network typically leads to lower stiffness and reduced load-bearing capacity [21]. Nonetheless, copolymers with QAUDMA-12 content below 20 wt.% retained acceptable mechanical performance, showing only moderate reductions in *FS* (up to 20%) and *E* (up to 10%) relative to the UDMA-based reference. The mechanical properties of the QAUDMA-12-based copolymers were benchmarked against those of a standard Bis-GMA/TEGDMA formulation (60:40 wt.%) characterized in our previous study [12]. From this comparison, it was found that all QAUDMA-12-based materials demonstrated superior or comparable mechanical properties, except the formulation containing 40 wt.% QAUDMA-12 exhibited a slight (7%) reduction in *E*.

Water sorption (*WS*) and solubility (*SL*) were assessed to determine the clinical suitability of QAUDMA-12-based copolymers in dental formulations. *WS* and *SL* increased with higher QAUDMA-12 content (Figure 6), likely due to the two quaternary ammonium groups in the QAUDMA-12 monomer, which increase hydrophilicity [22]. The ionic groups enhance moisture affinity, raising *WS*, while unreacted QAUDMA-12 residues, because of their hydrophilic and ionic properties, are more likely to leach from the polymer, increasing *SL*. Even at the lowest QAUDMA-12 level, *WS* rose by 8% and *SL* by 300% compared to the reference copolymer. However, all QAUDMA-12-modified formulations remained within ISO 4049 limits for dental polymer-based materials, which specify maximum values of 40 µg/mm^3^ for *WS* and 7.5 µg/mm^3^ for *SL* [18].

The antimicrobial activity of QAUDMA-12-based copolymers was evaluated against Gram-positive *Staphylococcus aureus* and *S. epidermidis* bacterial strains, Gram-negative *Escherichia coli* bacterial strains, and fungal *C. albicans* strains by quantifying the number of colonies adhered to the copolymer surface. Although they are not considered cariogenic strains, they were selected for other reasons. *S. aureus* and *E. coli* were selected as model bacterial strains, whereas *S. epidermidis* and *C. albicans* were selected as they naturally occur in oral microbiota.

In all tested strains, a concentration-dependent reduction in the number of adhered colonies was observed with increasing QAUDMA-12 content (Table 2), confirming that the quaternary ammonium groups play a critical role in antimicrobial performance [23]. Based on the data summarized in Table 2, a QAUDMA-12 content of 20 wt.% was identified as the minimum effective concentration for achieving substantial antimicrobial activity. At this level, reductions in microbial adherence reached approximately three orders of magnitude for *S. aureus*, two for *S. epidermidis*, and one for both *E. coli* and *C. albicans*. At lower QAUDMA-12 concentrations, the antimicrobial effect was notably less pronounced. Formulations with less than 20 wt.% QAUDMA-12 achieved only one order of magnitude reduction for *S. aureus* and *C. albicans*, while no statistically significant decrease in adhered colony number was observed for *S. epidermidis* and *E. coli*. These differences in antimicrobial activity among selected bacterial strains are due to differences in their cell wall structures. Quaternary ammonium salts exhibit the highest antibacterial activity against Gram-positive bacteria, as they possess a thick peptidoglycan layer rich in negatively charged components. It facilitates strong electrostatic interactions with the positively charged quaternary nitrogen, thereby disrupting the cytoplasmic membrane and causing bacterial cell death. On the contrary, Gram-negative bacteria have an additional outer membrane composed of lipopolysaccharide, which reduces the effectiveness of quaternary ammonium salts. Similarly, the outer membrane composed of ergosterol restricts the antifungal activity of quaternary ammonium compounds against *C. albicans* [24,25].

The observed reduction in the number of microbial colonies on the copolymer surfaces does not clearly indicate microbial cell death but rather indicates that the number of living microbial cells accumulated on the copolymer surfaces decreased. Therefore, the results should be interpreted as an evaluation of anti-colonization properties rather than as direct evidence of bactericidal or bacteriostatic effects.

Cytotoxicity of QAUDMA-12-based copolymers was evaluated using the L929 mouse fibroblast cell line. All QAUDMA-12-containing copolymers showed a reduction in cell viability greater than 70% as measured by Alamar Blue (Figure 7), which, according to ISO 10993-5, classifies them as noncytotoxic [19].

## 5. Conclusions

The research hypothesis that adjusting the QAUDMA-12 content would enable the development of antibacterial dental materials exhibiting an optimal balance of microbiological efficacy, biocompatibility, and physicochemical properties suitable for clinical application was confirmed.

The physicochemical properties of QAUDMA-12-based copolymers revealed that those with a QAUDMA-12 content below 30 wt.% meet the requirements for dental materials. They had a high degree of conversion, low water sorption and solubility, and mechanical properties higher than the properties of typical Bis-GMA/TEGDMA copolymers. The biological studies showed that the minimum QAUDMA-12 concentration providing sufficient antimicrobial activity was 20 wt.%. They also revealed that all tested formulations were non-toxic to the L929 mouse fibroblast cell line. Therefore, it can be concluded that the concentration of 20 wt.% QAUDMA-12 enables the production of non-toxic dental matrices with antimicrobial activity and the desired physicochemical properties.

This fundamental research led to the conclusion that it is possible to achieve dental composite restorative materials of enhanced antimicrobial activity. To finally assess the potential of QAUDMA-12-based dental composite clinical application, further research involving the dental composites composed of 20 wt.% QAUDMA-12-based matrix and various inorganic fillers should be used. Those studies should include an assessment of antibacterial activity against cariogenic bacterial strains (e.g., *S. mutans*), cytotoxicity using advanced cell lines (e.g., dental pulp cells), and a more detailed characterization of mechanical properties.

## Figures and Tables

**Table 1:** Sample name and composition.

Copolymer	Monomer Content (wt.%)			
	Bis-GMA	QAUDMA-12	UDMA	TEGDMA
40%QA12	40	40	0	20
30%QA12		30	10	
20%QA12		20	20	
10%QA12		10	30	
5%QA12		5	35	
2.5%QA12		2.5	37.5	
0%QA12		0	40	

**Table 2:** The number of microorganisms adhered to the studied copolymer surface. Lowercase letters indicate statistically significant (*p* ≤ 0.05) differences with a column (non-parametric Kruskal–Wallis with Mann–Whitney U post hoc test).

Copolymer	Number of Microorganisms Adhered to the Copolymer Surface (log(CFU/mL))							
	*S. aureus* (ATCC 25923)		*S. epidermidis* (ATCC 12228)		*E. coli* (ATCC 25922)		*C. albicans* (ATCC 2091)	
	Avg.	SD	Avg.	SD	Avg.	SD	Avg.	SD
40%QA12	2.59 ^a^ *	0.40	4.48 ^a–e^	0.28	0.00 ^a^ *	0.00	2.26 ^a^ *	0.19
30%QA12	3.64 ^b–e^	0.42	4.94 ^f–i^	0.26	5.01 ^b^ *	0.09	3.36 ^b^	0.41
20%QA12	3.89 ^f–i^	0.62	5.05 ^j–m^	0.36	5.59 ^c–e^	0.44	3.41 ^c^	0.36
10%QA12	5.83 ^b,f,j^	0.60	6.78 ^b,f,j,n^	0.25	6.11 ^f^	0.31	3.42 ^d^	0.23
5%QA12	5.82 ^c,g,k^	0.20	7.22 ^c,g,k,n^	0.18	6.41 ^c^	0.08	3.63 ^e^	0.18
2.5%QA12	5.84 ^d,h,l^	0.19	7.06 ^d,h,l^	0.66	6.43 ^d^	0.18	3.65 ^f^	0.17
0%QA12	6,74 ^e,i,j–l^	0.30	7.36 ^e,i,m^	0.53	6.70 ^e,f^	0.27	4.07 ^b–f^	0.30
control	10.02	0.38	10.52	0.24	9.98	0.18	7.53	0.33

* The result was statistically significantly different (*p* ≤ 0.05) compared to the results achieved by the remaining samples.

**Table 3:** The relative differences in the studied properties between the modified copolymers and the reference copolymer, indicating whether each parameter is higher (↑), lower (↓), or unchanged (–).

Property	Copolymer					
	40%QA12	30%QA12	20%QA12	10%QA12	5%QA12	2.5%QA12
degree of conversion (*DC*)	↑	↑	↑	↑	–	–
hardness (*HB*)	–	–	–	–	–	–
flexural strength (*FS*)	↓	↓	↓	↓	↓	–
flexural modulus (*E*)	↓	↓	↓	–	–	–
water sorption (*WS*)	↑	↑	↑	↑	↑	↑
water solubility (*SL*)	↑	↑	↑	↑	↑	↑
antibacterial activity *S. aureus*	↑	↑	↑	↑	↑	↑
antibacterial activity *S. epidermidis*	↑	↑	↑	–	–	–
antibacterial activity *E.coli*	↑	↑	↑	↑	–	–
antifungal activity *C. albicans*	↑	–	–	–	–	–
cytotoxicity L929	–	–	–	–	↑	↑

## Data Availability

The raw data supporting the conclusions of this article will be made available by the authors on request.

## Abbreviations

The following abbreviations are used in this manuscript:

Bis-GMA	bisphenol A glycerolate dimethacrylate
CQ	camphorquinone
DC	degree of conversion
DCRM	dental composite restorative materials
DMAEMA	2-dimethylaminoethyl methacrylate
E	flexural modulus
FS	flexural strength
FT-IR	Fourier Transform Infrared Spectroscopy
HB	hardness
*p*	significance level (statistical analysis)
PET	polyethylene terephthalate
QACs	quaternary ammonium compounds
QAMs	quaternary ammonium methacrylates
QAUDMA-m	quaternary ammonium urethane-dimethacrylate monomers
QAUDMA-12	quaternary ammonium urethane-dimethacrylate monomer with twelve methylene groups in N-alkyl chains
SD	standard deviation
SL	water solubility
TEGDMA	triethylene glycol dimethacrylate
UDMA	urethane-dimethacrylate monomer
WS	water sorption
